# Socioeconomic and sex inequalities in chronic pain: A population-based cross-sectional study

**DOI:** 10.1371/journal.pone.0285975

**Published:** 2023-05-25

**Authors:** Ana Maria Braga de Oliveira, Doralice Severo da Cruz Teixeira, Fabrício dos Santos Menezes, Amélia Pasqual Marques, Yeda Aparecida de Oliveira Duarte, Raquel Aparecida Casarotto

**Affiliations:** 1 Department of Physiotherapy, Federal University of Sergipe, Lagarto, Brazil; 2 Rehabilitation Sciences Program, School of Medicine, University of São Paulo, São Paulo, Brazil; 3 “Health, Well-being and Aging” (SABE) Study, School of Public Health, University of São Paulo, São Paulo, Brazil; 4 Department of Health Education, Federal University of Sergipe, Lagarto, Brazil; 5 School of Dentistry, University of São Paulo, São Paulo, Brazil; Marie Stopes International, PAKISTAN

## Abstract

**Objective:**

We investigated the impact of socioeconomic inequalities on chronic pain of older adults according to sex.

**Materials and methods:**

This population-based cross-sectional study used survey data from the 2015 cohort of the SABE Study (Saúde, Bem-estar e Envelhecimento), Brazil. Socioeconomic status was examined at individual level (educational attainment, financial independence, and race/skin color) and contextual level (Human Development Index). We analyzed the association between variables using the chi-square test and the Rao & Scott correction. Logistic regression models were adjusted for risk factors.

**Results:**

The study comprised 1,207 older adults representing 1,365,514 residents 60≥ years of age in the city of São Paulo. Chronic pain was more frequent in females (27.2%) than in males (14.5%) (p<0.001). Females evidenced the worst self-perception of pain, especially those of the most vulnerable socioeconomic strata. Social inequalities impacted chronic pain in different ways between sexes. Among females, unfavorable living conditions (OR = 1.59; 95%CI 1.07; 2,37) and Blacks/Browns females were most likely to have chronic pain (OR = 1.32; 95%CI 1.01; 1.74). Among males, only the individual aspects were significant for the occurrence of chronic pain, such as low educational attainment (OR = 1.88; 95%CI 1.16; 3.04) and insufficient income (OR = 1.63; 95%CI 1.01; 2.62).

**Discussion:**

The potential for inequality was greater for females than for males reflecting structural factors inherent in a highly unequal society. Conclusions: Equity-oriented health policies are critical to preventing pain in human aging.

## Introduction

Globally, the number of people 60 years and older is expected to increase to 2 billion by 2050 due to rapid population aging [1]. Among older adults, chronic pain is a common health condition associated with cognitive decline, dementia [2,3], and the risk of premature death [4,5]. Moreover, this multidimensional phenomenon leads to suffering, social isolation [4], negative impacts on health and quality of life [5,6], and high costs for healthcare services [4]. There is sufficient evidence about the relationship between chronic pain and individual variables [7]; however, contextual factors are still little examined in older adults based on sex.

The environment in which older adults live can affect chronic pain [8]. In low- and middle-income countries, the prevalence of chronic pain reaches 56.0–76.2% of the population [9,10], whereas it represents 19.0–20.4% in high-income countries [11,12]. Although chronic pain has been associated with socioeconomic status (SES) [13], this issue is not fully understood in highly unequal countries due to multiple underlying factors. In Brazil, for example, socioeconomic inequalities are not only related to sex, but also to the individuals’ race/skin color [14]. Structural racism in the country exposes black women and black men to a higher susceptibility to disease [15,16].

In parallel, pernicious sex inequalities in society could explain why chronic pain is more common in females [17,18] than males. Since, biological mechanisms and individual characteristics solely do not clarify the role of inequalities in later life and their cumulative effects on chronic pain, which depends on the characteristics of society. Based on these aspects, we hypothesized environmental factors influence chronic pain differently between sexes in highly unequal countries.

The growing population aging urged us to understand the role of inequalities on chronic pain to guide actions for pain prevention seeking to reduce its unequal burden in sexes. In parallel, Brazil presents social and sex inequalities due to high-income concentration, and one of the highest prevalences of chronic pain worldwide [10]. In 2016, the weekly working time on people care and/or household activities was 10.5 hours for men and 18.1 hours for women. The difference was also evident in income. While men earned approximately 2,306.00 reais, women earned 1,764.00 reais [19]. In studies dealing with chronic pain in Brazil, aspects such as being a woman [10,20,21] and having a low level of education in both sexes are mentioned as associated factors [22].

Investigating the epidemiology of pain, with particular attention to sex, race/skin color, and socioeconomic status, is critical because these factors may play an important role in the development, treatment, and management of pain [23,24]. Therefore, we investigated whether inequalities influence the occurrence of chronic pain in older adults according to sex, by assessing the self-reported pain intensity during daily activities across socioeconomic conditions.

## Materials and methods

This is a population-based cross-sectional study using data from the 2015 cohort of the SABE study (Saúde, Bem-estar e Envelhecimento). The SABE study is a population-based longitudinal research that provides information on the living and health conditions of older adults (60≥ years) in the urban area of the city of São Paulo, Brazil, since 2000. A detailed description of the study design and sampling procedure of the SABE study has previously been published [25,26]. In 2015, 1,224 older adults participated in the SABE study, representing 1,365,514 older adults ≥60 years of age. For the present study, 17 individuals were excluded because they did not provide information on the variable chronic pain. The sample consisted of 1,207 older adults people representing 1,353,333 older adults, residents of the city of São Paulo.

The selection of variables took into account the model established by the World Health Organization for actions towards social determinants of health [27] and the explanatory variables recognized in the scientific literature as factors associated with chronic pain [24]. The dependent variable was chronic pain (yes and no) with a duration of 3≥ months. We also assessed pain intensity during daily activities through a numerical pain scale, which measures the painful stimulus through numbers ranging from 0 (no impairment) to 10 (full impairment).

We investigated the role of socioeconomic inequalities in chronic pain at individual and contextual levels. Educational attainment (0–7 and 8 ≥ years) and financial independence (yes and no) were deemed as individual socioeconomic markers. Furthermore, self-reported race/skin color (white and black/brown) was assessed. Although race/skin color is a demographic variable, it is also considered a proxy for health inequalities in Brazil, a highly unequal country [28] which deals with ethnic-racial inequalities due to structural racism in its society. Furthermore, at a contextual level, the 2010 Human Development Index (HDI) was considered for the 31 districts of the city of São Paulo. The HDI is a globally recognized indicator, which makes it feasible to understand a local social reality by measuring three dimensions of human development: education, income, and life expectancy [29]. The Human Development Index ranges from 0 to 1, with higher values representing better human development [30]. In all analyzes, the HDI was classified as follows: first tertile: 0.680 to 0.768; second tertile: 0.777 to 0.822; and third tertile: 0.824 to 0.942. Thus, the individuals in the first tertile were in neighborhoods with the lowest level of human development.

The explanatory variables were age group (60–79 and 80≥ years) and labor force (active and inactive), being considered active subjects who still work, and inactive those who are retired. and behavioral and lifestyle variables, such as alcohol consumption (no and yes), smoking (no and yes), and physical activity (no and yes). Body Mass Index (BMI) was used to measure health status, with the following cut-off points for the older adults: underweight (23≤); eutrophic (23> and <28); overweight (28≥ and <30); and obese (30≥) [31].

In addition, cognitive function was analyzed as unimpaired and impaired through the Mini-Mental State Examination (MMSE), considering a score of 12 or lower as suggestive of cognitive impairment [32]. The older adults who had a performance equal to or greater than thirteen points were classified as unimpaired in terms of cognitive function. The Geriatric Depression Scale—short form (GDS-SF) was applied to determine the presence of depression. The older adults who scored 5≥ points were classified as depressive. Among the health conditions related to chronic pain, we also investigated the number of non-communicable diseases (0–1 and 2≥ illness) and the presence of sleep disorders (no and yes). In parallel, we investigated other explanatory contextual variables, such as participation in cultural activities (no and yes); difficulty accessing health services (no and yes); and the perception of neighborhood violence (never, sometimes, and always).

We performed stratified analyses to assess sex differences in pain perception of older adults. Data were described as frequencies for categorical variables and the mean and 95% confidence interval (CI) for continuous variables. The chi-square test with Rao & Scott correction was used to indicate the association between chronic pain and explanatory variables. Besides, we also carried out logistic regression models to calculate the odds ratio (OR) and its 95% CI. In multi-regression models, we investigated the effect of socioeconomic conditions (educational attainment, financial independence, race/skin color, and HDI) on chronic pain adjusted for risk factors, such as age group, cognitive function, depression, number of non-communicable diseases, sleep disorders, and smoking. Therefore, we assessed its accuracy using a goodness-of-fit test for logistic regression. All analyses considered the probabilistic sample of the SABE study and its sampling weights. Further, hypothesis tests were two-sided and a p-value < .05 indicated the statistical significance.

The Research Ethics Committee of the School of Medicine of the University of São Paulo (USP) approved this study (process number: 4.144.605), and we obtained written consent from all participants.

## Results

In this study, 1,207 older adults were eligible to participate in the research, which represents 1,365,514 residents 60≥ years in the city of São Paulo. Our sample presented the highest prevalence of women (Percentage_weighted_ = 56.1%; n = 783; n_weighted_ = 759,606.48), subjects aged 60–79 years (Percentage_weighted_ = 85.4%; n = 985; n_weighted_ = 1,155,154.65), white (Percentage_weighted_ = 52.7%; n = 624; n_weighted_ = 710,288.37), and with <7 years of schooling (Percentage_weighted_ = 68.1%; n = 833; n_weighted_ = 920,878.76). Therefore, chronic pain occurred in 41.7% of older adults (n = 521; n_weighted_ = 564,500.39), which was more common in females (27.2%) than in males (14.5%) (p<0.001) (Table 1).

**Table 1:** Baseline sample characteristics by sex and self-reported chronic pain in adults aged 60 and older. SABE Study (Saúde, Bem-estar e Envelhecimento), Brazil, 2015.

Variables	Female						Male					
	No			Yes			No			Yes		
	%**	n^†^	n^§^	%*	n^†^	n^§^	%**	n^†^	n^§^	%*	n^†^	n^§^
Sociodemographic Conditions												
Age group												
60–79 years	43.2	327,929	316	41.4	314,488	312	57.7	342,434	241	28.7	170,303	116
80≥ years	8.4	63,481	82	7.1	53,708	73	9.3	54,989	47	4.4	26,000	20
Educational Attainment												
0–7 years	35.0	265,803	277	35.1	266,620	281	40.3	239,457	172	25.1	148,999	103
8≥ years	16.6	125,606	121	13.3	100,720	103	26.6	157,966	116	8.0	47,304	33
Labor force												
Economically Inactive	38.9	292,664	301	37.5	282,226	294	33.9	200,628	144	21.2	125,738	85
Economically Active	12.4	93,696	92	11.2	84,539	90	33.0	195,417	143	11.9	70,565	51
Financial Independence												
No	23.6	176,246	176	26.5	197,751	202	26.3	154,392	116	18.4	107,852	73
Yes	27.8	207,477	213	22.1	164,869	177	40.6	238,362	168	14.8	87,121	62
Race/skin color												
Black/Brown	23.0	173,988	180	25.2	191,100	197	31.1	183,469	136	15.0	88,316	64
White	28.5	216,136	216	23.3	176,028	187	35.6	210,137	149	18.3	107,987	72
Behavior & Lifestyle												
Alcohol consumption												
No	46.6	353,392	359	44.9	340,248	356	42.9	254,577	185	24.0	142,311	101
Yes	5.0	37,635	38	3.5	26,701	27	24.1	142,846	103	9.1	53,992	35
Smoking												
No	31.3	237,967	242	29.4	223,178	232	24.6	145,742	102	6.8	40,627	28
Yes	20.2	153,443	156	19.1	145,019	153	42.4	251,681	186	26.2	155,676	108
Physical Activity												
No	24.8	188,239	197	24.8	188,382	202	46.1	273,915	197	24.9	147,773	103
Yes	26.8	203,171	201	23.7	179,815	183	20.8	123,508	91	8.2	48,531	33
Health Status												
Body Mass Index												
Eutrophic	19.7	135,851	142	15.9	109,467	114	31.4	168,022	122	13.8	73,717	51
Underweight	5.1	34,924	37	4.6	31,892	33	11.8	62,820	46	5.7	30,718	21
Overweight	6.9	47,376	46	7.9	54,199	54	8.9	47,763	35	4.5	24,055	16
Obese	20.4	140,637	137	19.5	134,528	138	16.4	87,621	59	7.4	39,739	28
Cognitive Function Impairment												
No	44.9	341,237	338	41.6	316,248	322	59.9	355,569	254	28.1	166,838	113
Yes	6.6	50,172	60	6.8	51,949	63	7.0	41,854	34	5.0	29,466	23
Depression												
No	43.0	318,556	322	35.6	264,287	272	60.9	354,410	254	26.7	155,425	106
Yes	8.6	63,967	63	12.8	94,665	101	6.0	35,029	28	6.4	36,960	27
Number of Non-communicable Diseases												
0–1	19.0	143,979	143	13.5	102,246	105	36.3	215,542	160	13.8	82,017	59
2 ≥	32.6	247,431	255	35.0	265,951	280	30.6	181,882	128	19.3	114,287	77
Sleep Disorders												
No	29.3	222,481	229	21.7	164,954	172	49.8	295,435	210	21.7	128,981	87
Yes	22.3	168,929	169	26.7	202,269	212	17.2	101,988	78	11.3	67,323	49
Contextual Factors												
Cultural Activities												
No	20.3	152,945	163	27.9	210,445	224	39.2	230,726	169	22.8	134,484	93
Yes	31.0	233,151	227	20.8	156,608	159	27.5	161,991	115	10.5	61,819	43
Difficulty of Access to Health Services												
No	31.0	235,173	244	26.6	201,785	212	47.6	281,296	199	22.0	129,852	87
Yes	20.6	156,236	154	21.9	166,412	173	19.4	114,680	88	11.1	65,356	48
Local Human Development Index												
3rd tertile	16.9	125,063	128	11.5	85,471	90	25.0	147,182	103	10.4	61,335	41
2nd tertile	16.9	125,759	131	17.1	127,229	136	20.6	121,355	93	12.0	70,772	51
1st tertile	17.8	132,056	129	19.8	146,790	149	21.3	125,541	89	10.7	62,990	43
Perception of violence in their neighborhoods												
Never	27.7	207,250	208	22.8	171,102	188	44.9	259,640	193	20.1	116,355	82
Sometimes	17.6	131,668	135	14.9	111,957	113	15.3	88,600	63	8.9	51,755	35
Always	6.1	45,839	45	10.9	81,381	78	6.5	37,599	25	4.3	24,957	17

*Statistically significant.

** Weighted proportions in percentage based on the complex sample design and sampling weights.

^†^ Weighted sample size.

^§^ Original cases.

In the univariate analysis, we found differences between sexes in factors associated with chronic pain. For females, the highest probability of chronic pain was observed in the following variables: race/skin color, and sleep disorders. For males, the factors associated with chronic pain were low educational attainment, professional inactivity, and smoking. Financial independence, depression, and the number of non-communicable diseases affected both sexes; however, contextual factors impacted chronic pain exclusively in females. For example, the lowest tertile of HDI and the worst perception of neighborhood violence increased the likelihood of chronic pain by 63% and 115%, respectively (Table 2).

**Table 2:** Univariate analysis of elderly subjects by sex and chronic pain self-reported. SABE Study (Saúde, Bem-estar e Envelhecimento), Brazil, 2015.

Variables	Female		Male	
	OR (CI 95%)	P-value^a^	OR (CI 95%)	P-value^a^
Sociodemographic Conditions				
Age group		0.556		0.886
60–79 years	Ref.		Ref.	
80≥ years	0.88 (0.58; 1.34)		0.95 (0.47; 1.91)	
Educational Attainment		0.196		***0*.*002*** *
8≥ years	Ref.		Ref.	
0–7 years	1.25 (0.89; 1.76)		**2.08 (1.31; 3.29)**	
Labor force		0.684		***0*.*011*** *
Economically Active	Ref.		Ref.	
Economically Inactive	1.07 (0.77; 1.48)		**1.74 (1.14; 2.65)**	
Financial Independence		*0*.025*		***0*.*006*** *
Yes	Ref.		Ref.	
No	**1.41 (1.05; 1.91)**		**1.91 (1.20; 3.04)**	
Race/skin color		*0*.030*		0.783
White	Ref.		Ref.	
Black/Brown	**1.35 (1.03; 1.77)**		0.94 (0.59; 1.50)	
Behavior & Lifestyle				
Alcohol consumption		0.277		0.115
No	Ref.		Ref.	
Yes	0.74 (0.42; 1.28)		0.68 (0.41; 1.10)	
Smoking		0.964		***0*.*003*** *
Never	Ref.		Ref.	
Yes	1.01 (0.72; 1.41)		**2.22 (1.31; 3.76)**	
Physical Activity		0.438		0.200
Yes	Ref.		Ref.	
No	1.13 (0.83; 1.55)		1.37 (0.84; 2.23)	
Health Status				
Body Mass Index		0.506		0.974
Eutrophic	Ref.		Ref.	
Underweight	1.13 (0.68; 1.88)		1.11 (0.56; 2.22)	
Overweight	1.42 (0.87; 2.31)		1.15 (0.61; 2.16)	
Obese	1.19 (0.83; 1.71)		1.03 (0.56; 1.91)	
Cognitive Function Impairment		0.545		0.209
No	Ref.		Ref.	
Yes	1.12 (0.78; 1.60)		1.50 (0.79; 2.84)	
Depression		*0*.001*		***0*.*007*** *
No	Ref.		Ref.	
Yes	**1.78 (1.27; 2.51)**		**2.41 (1.25; 4.62)**	
Number of Non-communicable Diseases		*0*.031*		***0*.*017*** *
0–1	Ref.		Ref.	
2≥	**1.51 (1.04; 2.21)**		**1.65 (1.10; 2.49)**	
Sleep Disorders		*0*.001*		0.069
No	Ref.		Ref.	
Yes	**1.61 (1.23; 2.12)**		1.51 (0.97; 2.36)	
Contextual Factors				
Cultural Activities		*<0*,001*		0.084
Yes	Ref.		Ref.	
No	**2.05 (1.50; 2.79)**		1.53 (0.94; 2.48)	
Difficulty of Access to Health Services		0.119		0.407
No	Ref.		Ref.	
Yes	1.24 (0.95; 1.63)		1.23 (0.75; 2.04)	
Local Human Development Index		*0*.020*		0.432
3^rd^ tertile	Ref.		Ref.	
2^nd^ tertile	**1.48 (1.02; 2.14)**		1.40 (0.84; 2.32)	
1^st^ tertile	**1.63 (1.13; 2.34)**		1.20 (0.72; 2.02)	
Perception of violence in their neighborhoods		*0*.003*		0.420
Never	Ref.		Ref.	
Sometimes	1.03 (0.73; 1.45)		1.30 (0.77; 2.22)	
Always	**2.15 (1.36; 3.41)**		1.48 (0.71; 3.08)	

Abbreviations: OR: Odds ratio; IC: Confidence interval.

^a^ Chi-square test and the Rao & Scott correction.

* Statistically significant.

Multiple regression analysis showed that social inequalities impacted chronic pain in different ways between sexes. Among females, unfavorable living conditions increased the probability of chronic pain by 54% and 59%, respectively, in the lowest HDI tertiles, revealing a negative dose-response relationship. Additionally, black/brown females were more likely (32%) to report chronic pain than white females. Among males, only the individual aspects were significant for the occurrence of chronic pain. Low educational attainment and financial deficiency increased the odds of males reporting chronic pain by 88% and 63%, respectively. We adjusted all models for age group, cognitive function, depression, amount of non-communicable diseases, and sleep disorders (Table 3).

**Table 3:** Multiple logistic regression analysis investigating the association between chronic pain and socioeconomic variables. SABE Study (Saúde, Bem-estar e Envelhecimento), Brazil, 2015.

Variables	Female^a^	Male^b^
	OR (CI 95%)	OR (CI 95%)
Socioeconomic Factors		
Educational Attainment		
8≥ years	Ref.	Ref.
0–7 years	1.07 (0.75; 1.54)	**1.88 (1.16; 3.04)**
Financial Independence		
Yes	Ref.	Ref.
No	1.19 (0.86; 1.65)	**1.63 (1.01; 2.62)**
Human Development Index		
3^rd^ tertile	Ref.	Ref.
2^nd^ tertile	**1.54 (1.05; 2.25)**	1.49 (0.88; 2.52)
1^st^ tertile	**1.59 (1.07; 2.37)**	1.13 (0.67; 1.92)
Race/skin color		
White	Ref.	Ref.
Black/Brown	**1.32 (1.01; 1.74)**	0.77 (0.49; 1.22)
Adjustment Variables		
Age group		
60–79 years	Ref.	Ref.
80≥ years	0.97 (0.61; 1.52)	0.89 (0.44; 1.82)
Cognitive Function Impairment		
No	Ref.	Ref.
Yes	0.91 (0.59; 1.41)	1.49 (0.77; 2.89)
Depression		
No	Ref.	Ref.
Yes	**1.67 (1.13; 2.45)**	**1.99 (1.02; 3.91)**
Number of Non-communicable Diseases		
0–1	Ref.	Ref.
2 ≥	1.42 (0.95; 2.12)	**1.58 (1.02; 2.45)**
Sleep Disorders		
No	Ref.	Ref.
Yes	1.34 (0.99; 1.82)	1.35 (0.84; 2.18)

Abbreviations: OR: Odds ratio; IC: Confidence interval.

* Statistically significant.

^a^ The goodness-of-fit test for logistic regression models in weighted samples indicated the model accuracy (p-value = 0.115). For females, smoking has been included only for model adjustment purposes (OR = 0.95; CI 95% = 0.68; 1.32; P-value = 0.755).

^b^ Females and males have evidenced different associated factors. Therefore, the multi-regression model did not include smoking to present a better precision (p-value = 0.159).

### Scores of self-reported pain intensity during daily activities

**Fig 1:**
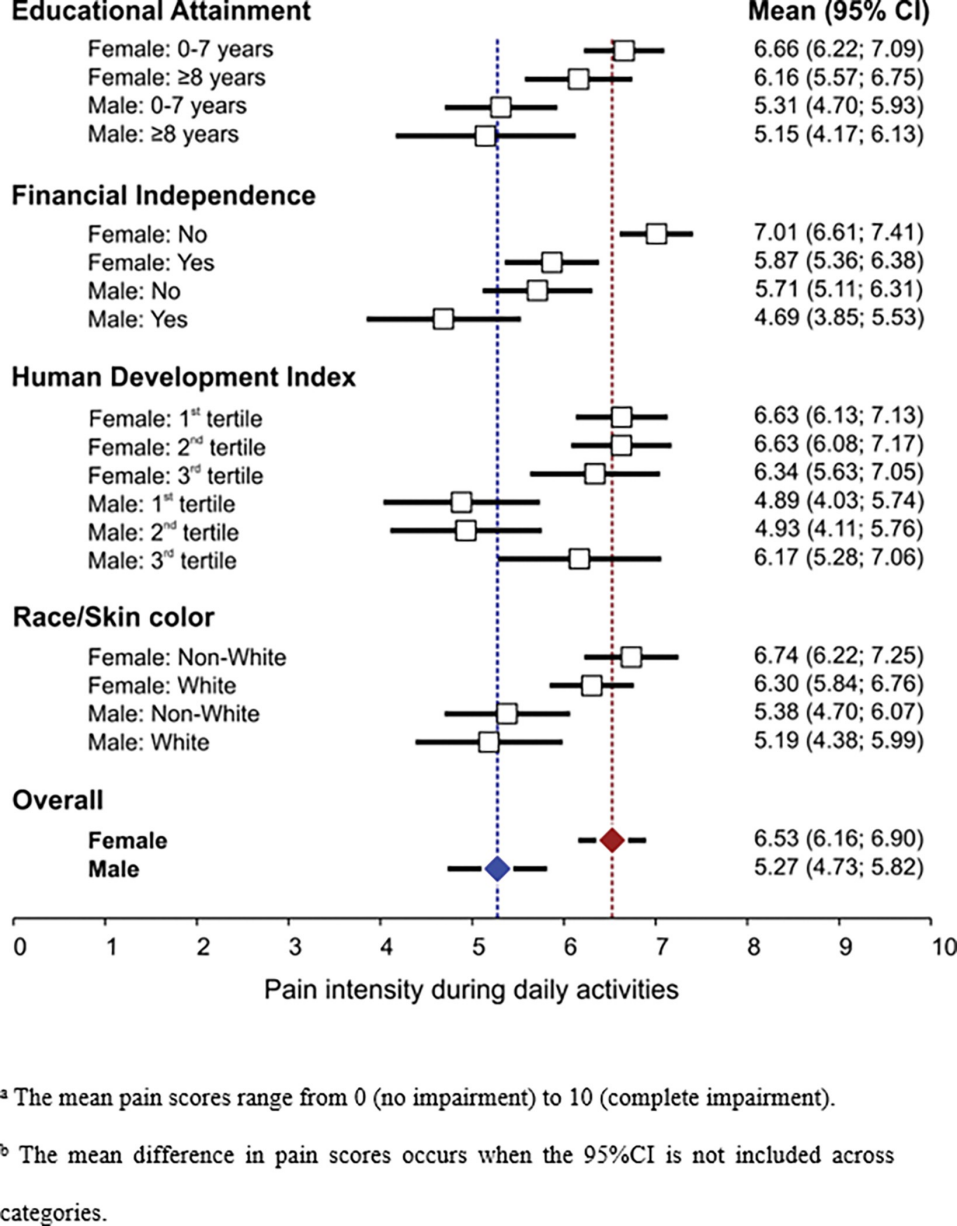
Socioeconomic status and scores of intensity of self-reported pain in daily activities among older adults by sex. SABE Study (Saúde, Bem-estar e Envelhecimento), Brazil, 2015.

In general, our data revealed that females (6.53; 95%CI = 6.16–6.90) had higher pain intensity scores than males (5.27; 95%CI = 4.73–5.82). Likewise, women evidenced the worst pain scores in educational attainment, financial independence, HDI, and race/skin color. Of note, females with financial deficiency had the worst self-perception of pain (7.01; 95%CI = 6.61–7.41) than males (4.69; 95%CI = 3.85–5.53) and females (5.87; 95%CI = 5.36–6.38) with sufficient resources (Fig 1). Therefore, we observed sex inequalities in pain scores across socioeconomic conditions, especially in the most vulnerable socioeconomic strata, which is consistent with the results of chronic pain measured with a qualitative approach.

## Discussion

To our knowledge, this is the first population-based study that highlights the differential role of socioeconomic inequalities in chronic pain in older adults, according to sex. In the female group, chronic pain was related to socioeconomic inequality factors, associated with living conditions and race/skin color. Our analysis has demonstrated a dose-response relationship between individuals living in areas with low HDI and presenting chronic pain, suggesting a social gradient when it comes to chronic pain in females. On the other hand, in males, the probability of chronic pain was associated solely with individual aspects. We observed that females, in general, seem to be more affected by chronic pain, especially within the most vulnerable social strata. This implies that chronic pain-related differences between sexes may not be based exclusively on biological traits, that is, it is also impacted by structural factors inherent to highly unequal societies such as low- and middle-income countries.

Contextual aspects impacted the presence of chronic pain exclusively in female individuals. Although the general living conditions of Brazilians have improved due to social policies such as “Bolsa Família” [33] during the study period, the opportunities do not appear to have been evenly distributed between males and females. This reflects the fragility of women-oriented social programs and reveals traits of a society in which females are still regular victims of violence [34]. Around the world, approximately 1 in 3 females are subjected to physical and/or sexual violence by an intimate partner or sexual violence by a non-partner in their lifetime [35]. Violence is also prevalent in older adults females [36] and negatively impacts their health condition, since such violent episodes tend to result in pain syndromes [37].

Regarding individual aspects, our findings show that black/brown females are more likely to develop chronic pain. Race/skin color is an important aspect affecting health, mainly due to racism [38]. In a longitudinal study, the presence of pain was associated with females victims of racism [39]. Likewise, blacks/browns females reported receiving less information from health care services about the effects of analgesics such as opioids [40], reinforcing the evidence of racial disparities in health care [41]. In this scenario, black women are doubly affected by racism and gender bias [42]. In addition, difficulties accessing health care services occur more commonly among females and black skin color individuals [43], and reducing barriers to accessing health care services is important for improving pain management [44].

Among males, the factors influencing chronic pain were solely those of individual character and related to low educational attainment and insufficient income. Income and schooling may reflect better health conditions [38,45]. Low-schooling individuals reported more pain than the higher-schooling ones. Likewise, in a longitudinal study, higher-income respondents were less likely to have pain than the low-income population [46]. In parallel, African Americans and individuals who were in the lower wealth quartile reported more disabilities related to pain [47].

Concerning pain intensity, females scored higher than males, which reinforces the evidence that pain is more intense in females [48]. Regarding pain scores and socioeconomic conditions, blacks/browns females and females with financial insufficiency reported the highest pain intensities. Some aspects are important to understand this disparity. Differences in pain perception concerning race/skin color are multifactorial and include socioeconomic factors [7]. Likewise, racism contributes to the severity of pain [39]. Moreover, job opportunities are not equal for males and females. Therefore, access to income generation sources is unequal. For example: in Brazil, although females present higher educational attainment than males, they receive, proportionally, lower wages. Additionally, in most cases, the lowest-wage activities are left to be carried out by black or brown race/skin color individuals and by females [19]. Therefore, in Brazil, females and blacks evidence the highest socioeconomic disadvantages [14].

Unfavorable socioeconomic factors, such as low income and low schooling, are considered predictors of the development of chronic pain [24]. Similarly, in the present article, subjects with the worst socioeconomic conditions were more likely to be affected by chronic pain. There are contrasting views in the literature regarding race/skin color [24]. Studies report higher pain intensity in black participants than in white individuals [49] but investigations have found no association between race and the presence of chronic pain [47]. Although our data suggest that race/skin color is an associated factor for chronic pain, further studies are needed to clarify this issue.

A negative correlation was observed between pain intensity and schooling, that is, the higher the educational attainment, the lower the intensity of pain. Other studies have shown similar results [46,50]. In general, people with higher educational levels tend to have better jobs and, therefore, better access to health services, which contributes to more adequate pain care [45]. The impact of education on health is at grassroots level. It leads to better general self-awareness about personal health care and makes health care more accessible [51].

Several studies report the highest prevalence and severity of chronic pain in females. Although there is insufficient information on sex differences in pain perception. There is biological evidence, such as the role of hormones [24]. Based on our findings, we hypothesized that underprivileged females are more likely to present chronic pain with greater intensity in unfavorable socioeconomic conditions, especially in structurally unequal countries such as Brazil. Thus, although both females and males reported pain, socioeconomic aspects differed between them, suggesting further studies to examine these sex differences in chronic pain.

Implementing policies to promote sex equality may help to change this scenario. As shown in this article, contextual aspects have an influence on the occurence of chronic pain, especially in women. Consequently, to remain healthy, it is necessary to mediate between the different sectors of society, with the empowerment of women, cultural changes that allow to combine work, family, and motherhood with the division of tasks between men and women, so that, for example, domestic activities are not only the responsibility of women.

This research presents limitations. We did not assess marital status or unpaid labor in our cross-sectional study in which associations occur, but no cause-and-effect relationships are established. Although there is a subjective component to pain assessment, the report of individuals on their pain should be recognized and respected once it means empowering individuals toward self-care. Notwithstanding, our method to measure pain is widely used in the literature [52]. Thus, we used well-designed population-based survey data, which makes it possible to perform an analysis stratified by sex and generalize the results to the 1,365,514 older adults, residents of the city of São Paulo.

## Conclusions

Chronic pain can be a social issue, resulting from inequalities related to an individual’s living, working, and aging conditions. In high-income concentration countries, the potential for inequality factors can be as critical as biological aspects. This study has identified differences in the influence of inequalities on the development of chronic pain in older adults, showing higher prevalence among females resulting from macro-social or structural aspects of society. Public policies must consider sex unevenness to reduce social inequalities and promote equity in comprehensive care policies for the population of older adult. Through this, the impacts of chronic pain on health systems are expected to be reduced and equal opportunities for females and males shall be provided, so that healthy aging and quality of life can be reached by both, male and female, equally.

## Supporting information

S1 Data set (XLSX)
